# Low expression of PRMT5 in peripheral blood may serve as a potential independent risk factor in assessments of the risk of stable CAD and AMI

**DOI:** 10.1186/s12872-019-1008-4

**Published:** 2019-01-31

**Authors:** Buchuan Tan, Qian Liu, Liping Yang, Yushuang Yang, Dongna Liu, Long Liu, Fanbo Meng

**Affiliations:** 1 China-Japan Union Hospital of Jilin University, Changchun, China; 2 First Hospital of Hebei Medical University, Shijiazhuang, Hebei China; 3 Peking University Shenzhen Hospital, Shenzhen, China; 4 Cardiology Department of the China-Japan Union Hospital of Jilin University, 126, Xiantai Street, Changchun City, 130033 NO China

**Keywords:** PRMT5, Stable CAD, AMI, Independent risk, Peripheral blood, Genetic marker

## Abstract

**Background:**

Protein arginine methyltransferases (PRMTs) can catalyse the methylation of arginine and participate in many important cellular reaction processes. The purpose of this research is to determine whether the expression levels of the PRMT5 gene in peripheral blood can be used as a biomarker for predicting the risk of Acute Myocardial Infarction (AMI).

**Methods:**

In this research, peripheral blood was collected from 91 patients with AMI and 87 patients with stable coronary artery disease (CAD). Real-time fluorescent quantitative PCR was performed to measure the expression levels of the PRMT5 gene at the mRNA level, and a western blot analysis was performed to measure the expression levels of the PRMT5 gene at the protein level.

**Results:**

The results indicate that at both the RNA and protein levels, the expression levels of the PRMT5 gene in peripheral blood from patients with AMI are significantly lower than those in peripheral blood from patients with stable CAD (Z = − 4.813, *P* = 0.000). The low expression of the PRMT5 gene is relevant to the Gensini score of the coronary artery (rs = − 0.205, *P* = 0.015) but is irrelevant to the serum level of blood lipids, level of cardiac troponin (rs = − 0.125, *P* = 0.413) and time intervals of occurrence (rs = − 0.146, *P* = 0.211). Patients who have a low PRMT5 expression in the peripheral blood are 5.472 times more likely to suffer from AMI than other patients.

**Conclusion:**

Compared to stable CAD patients, AMI patients have a lower expression of the PRMT5 gene in their peripheral blood. Patients who have low PRMT5 gene expression in the peripheral blood are more likely to suffer from AMI than those with stable CAD. A low expression of the PRMT5 gene serves as an independent risk factor for the occurrence of AMI.

## Background

Myocardial infarction (MI) is a global disease characterized by a high incidence and high mortality. More than 3 million people were newly diagnosed with ST-segment Elevation Myocardial Infarction (STEMI) and more than 4 million people were newly diagnosed with Non-ST-segment Elevation Myocardial Infarction (NSTEMI) worldwide. The incidence of myocardial infarction is relatively higher in developed countries and is rapidly increasing in developing counties, including China [1]. Relevant epidemiological and basic science studies indicate that lifestyle factors and genetic factors jointly promote the progression of coronary atherosclerosis and the occurrence of myocardial infarction [1–4]. An investigation involving over 15,000 MI patients was conducted by INTERHEART and indicated that most MI cases were caused by interactions among risk factors (i.e., smoking, dyslipidaemia, hypertension, abdominal obesity, diabetes, etc.) and susceptibility genes, resulting in coronary atherosclerosis [4, 5]. Coronary heart disease is a complex genetic disease, and important inducing genes and susceptibility genes have been identified through many genetic studies using modern genetic technologies [4].

Numerous studies have indicated that the gene expression levels in peripheral blood can reflect changes in and the progression of complex cardiovascular diseases, thus serving as a very significant biomarker for examining and diagnosing cardiovascular diseases [6, 7]. Multiple studies have verified that gene module analyses can be used as a biomarker of disease progression or disease prognosis, and modular analyses of gene expression can be used to determine the prognosis of patients with breast cancer independent of breast cancer grading [8]. The mRNA co-expression modules of AKT1, BRCA1, CCDC134, UBD and ZIC2 in peripheral blood can be used as biomarkers describing the progression of early-onset schizophrenia [9]. The expression level of single genes at the mRNA level in peripheral blood can also be used as a genetic marker for the assessment of disease progression, and the overexpression of KIAA0101 at the mRNA level can be used as a predictor of the invasion and progression of liver cancer [10], while the expression of KLKB1 at the mRNA level in monocytes in peripheral blood can be used as a molecular marker for the diagnosis and identification of chronic lymphocytic leukaemia patients and non-leukaemia patients [11]. The expression level of CD36 at the mRNA level in monocytes in peripheral blood may mark the progression of coronary heart disease [12], and the expression level of AdipoR2 at the mRNA level in peripheral blood is correlated with the progression of coronary atherosclerosis [13].

A pathological study investigating myocardial infarction indicated that plaque rupture and subsequent thrombosis serve as the most important mechanisms of AMI [14, 15]. In atherosclerosis, the imbalance in endothelial function can promote the occurrence of inflammatory activity [16]. The endothelium can release adhesion molecules to absorb leucocytes and penetrate the intima, causing lipid deposition on the vessel wall and vascular inflammation. The inflammatory reaction can further promote phagocytosis of lipids by macrophages and, thus, the formation of foam cells. T-cells enter the intima and secrete cytokines that can enhance the inflammatory response and promote the migration and proliferation of smooth muscle cells in the intima [17]. Furthermore, the inflammatory activity can weaken the fibrous cap of the atheromatous plaque and cause the occurrence of thrombosis, thus resulting in the occurrence of AMI [17, 18].

The protein arginine methyltransferase (PRMT) family participates in many cellular responses, including the inflammatory response. The inflammatory response can cause various diseases related to inflammation or autoimmune diseases, including rheumatic arthritis, Alzheimer’s disease, systemic lupus erythaematosus, asthma, atherosclerosis, cancer, ischaemic heart disease, etc. [19]. PRMT5 performs the function of regulating the on-off action of the inflammatory response [19]. The results of an analysis of the differential gene expression profile in peripheral blood in myocardial infarction patients previously conducted by this research group indicate that compared to stable CAD patients, AMI patients had a differential low expression of the PRMT5 gene in their peripheral blood, and the expression level was 0.642 lower. Therefore, by expanding the sample size of the clinical data, this research group further investigated whether the PRMT5 gene, which is related to the inflammatory response, is correlated with AMI, assessed the value of the PRMT5 gene in the diagnosis of AMI and investigated whether the PRMT5 gene can be used as a biomarker in assessments of the risk of AMI.

## Method

### Research subjects

All studied subjects were recruited among the patients admitted to the Department of Cardiovascular Medicine, China-Japan Union Hospital of Jilin University between April 2016 and September 2016 and subjected to coronary angiography. The patients definitively diagnosed with AMI were selected based on the global universal definition of myocardial infarction issued in 2012 [20] and included in the AMI Group, while patients with Stable CAD [21] were selected and included in the control group (1. narrowing of ≥50% in the left main coronary artery and ≥ 70% in one or several major coronary arteries, and 2. brief duration of discomfort no longer than 10 min in most cases). The exclusion criteria used in this research were as follows: 1. myocardial infarction related to Percutaneous Coronary Intervention (PCI) or Coronary Artery Bypass Grafting (CABG); 2. MI type II, i.e., secondary myocardial infarction related to an imbalance between the supply and demand of blood or myocardial infarction caused by an increase in the catecholamine level or coronary spasm; 3. myocardial infarction accompanied by cardiac surgery or non-cardiac surgery; 4. multi-factor or uncertain myocardial injuries caused by uncertain diseases, such as severe heart failure, stress-induced cardiomyopathy, severe pulmonary embolism or pulmonary hypertension, sepsis, critical diseases, kidney failure and severe nervous system diseases, such as stroke, subarachnoid haemorrhage, etc.; 5. immune system diseases and (or) the use of hormones; 6. a history (active or latent) of tuberculosis or evidence of tuberculosis; 7. chronic or recurrent infectious diseases or a medical history of such diseases; 8. complications of severe infectious diseases and malignant tumour or suspected or confirmed immunodeficiency; and 9. incomplete clinical data, including blood lipid level, fasting blood glucose level, sitting blood pressure, body mass index (BMI), family medical history of coronary heart disease, smoking history, coronary arteriography data and information about complications of other clinical diseases (such as hypertension, diabetes), or incomplete coronary angiography data.

### Research methods

#### Peripheral blood collection, Total RNA extraction and cDNA synthesis

In total, 4 ml of peripheral venous blood were collected from each subject, followed by total RNA extraction using a total RNA extraction reagent kit (RNA Simple Total RNA Kit, Tiangen Biotech (Beijing) Co., Ltd., Beijing) according to the company’s instructions. The incipient long-time (≥20 min) rest pain of the patients with AMI in the prior week was considered the time of onset, and the interval from the onset of AMI in the patients in the AMI Group to the time of blood collection was considered the time interval of occurrence. The quality and concentration of the RNA were tested using 1.5% agarose electrophoresis and an ultraviolet spectrophotometer (Nanodrop 2000). Qualified RNA samples should meet the following requirements: the value of A260/A280 should range from 1.9 to 2.1, and the value of A260/A230 should be greater than 2. In the agarose gel electrophoresis, there should be bright 28S and 18S rRNA bands, and the brightness of the 28 s rRNA band should be approximately twice the brightness of the 18 s rRNA band. Then, 1 μg of qualified total RNA was subjected to reverse transcription conducted by using a reverse transcription reagent kit (TOYOBO Rever Tra Ace qPRC RT kit, Shanghai).

#### Real-time quantitative PCR analysis

A SYBR real-time quantitative PCR reagent kit (SYBR Premix Ex Taq TM, TaKaRa, Dalian) was used for the PCR amplification. In total, a 20 μl-reaction system and an Mx3005P real-time quantitative PCR system (Strata Gene) were used for the amplification. The conditions of the reaction were as follows: 1 min of pre-degeneration at 95 °C, 5 s of degeneration at 95 °C, and 40 cycles of 30 s of annealing at 60 °C. After the reaction finished, the dissociation curve and amplification curve within the temperature range of 60 °C and 95 °C were recorded, and the reaction products were stored at 4 °C for use. The cycle thresholds (Ct) acquired for each sample were expressed as the relative expression quantity 2^-△Ct^ (△Ct = Target Gene Ct Value - Reference Gene Ct Value). See Table 1 for information regarding the primer sequences.

**Table 1:** Sequences of the primers used for real-time RT-PCR

Genes		Genes Primer Sequence (5′-3′)
PRMT5	F^a^	TGTAGGGAGAAGGACCGTGA
	R^b^	ATGGCTGAAGGTGAAACAGG
GAPDH	F^a^	ACGGATTTGGTCGTATTGGGCG
	R^b^	CTCCTGGAAGATGGTGATGG

F^a^, sequence from sense strands

R^b^, sequence from anti-sense strands

#### Western blot analysis

After using RIPA to collect the peripheral blood leukocytes from the patients, the supernatants were centrifuged and placed in a 98 °C water bath for 10 min. Then, 5 x Loading buffer was added, and 40 μg of protein were applied to an SDS-page gel; electrophoresis was performed at 80 V for 30 min, followed by electrophoresis at 100 V for 60 min. According to the BD semi-dry instruction manual, the proteins were transferred to a PVDF membrane and incubated with the primary antibody overnight in 4 °C; then, the membranes were incubated with the secondary antibody at room temperature for 2 h, and analysed using a chemiluminescence imaging system.

### Statistical analysis

All data were statistically analysed with SPSS24.0 software. The statistical analysis of the relative expression levels in the AMI Group (compared with the control group) was conducted by using the 2^-△△Ct^ method [22] as follows: △△Ct = △Ct of the AMI Group - △Ct of the stable CAD Group. Both independent t-tests and rank-sum tests were applied to compare the groups. The x^2^ test was applied to the enumeration data to analyse the inter-group differences. A binary logistic regression analysis was carried out to analyse the relevant risk factors of AMI. An analysis of the correlations among the relative expression level of the PRMT5 gene, cardiac troponin I level and Gensini score was conducted by using a double-variable correlation analysis. *P*-values less than 0.05 were considered significant.

### Coronary Score

Coronary artery score equals the sum of all segment scores (each segment score equals segment tor multipl weighting facied by a severity score). Severity scores assigned to the specific percentage luminal diameter reduction of the coronary artery segment are 32 for 100%, 16 for 99%, 8 for 90%, 4 for 75%, 2 for 50%, and 1 for 25%.

## Results and discussion

### Clinical data analysis

The results of the clinical data analysis of the research objects indicate that there is no significant difference between the two groups in the aspects of age, sex, BMI, history of hypertension, family history of coronary heart disease, systolic pressure, diastolic pressure, serum triglyceride (TG) and high-density lipoprotein cholesterol (HDL-C). There were no significant differences regarding the use of oral antiplatelet agents, statins, beta-blockers, or CCB. The serum levels of total cholesterol (TC) and low-density lipoprotein cholesterol (LDL-C) in the patients in the AMI Group are obviously higher than those in the patients in the stable CAD Group, and this difference is statistically significant. AMI patients had more PCI rate, more use of Medications(ACE-I/ARB,), and worse EF values. See Table 2 for details.

**Table 2:** Comparison of Clinical Data between the AMI Group & stable CAD Group

Categories of Data	The AMI Group	The stable CAD Group	t/x^2^/z	*P* Value
	*N* = 91	*N* = 87		
Age (years old)	63.51 ± 10.506	61.9 ± 9.077	1.095	0.275
Sex			1.185	0.276
Male	59(64.8%)	63(72.4%)		
Female	32(35.2%)	24(27.6%)		
BMI (Kg/m^2^)	24.728(22.477–27.357)	25.626 ± 3.188	−1.711	0.087
Hypertension	46(50.5%)	44(50.6%)	0.000	0.997
Family Medical History	7(7.7%)	4(4.6%)	0.718	0.397
Systolic Pressure (mmHg)	135.08 ± 24.740	136.38 ± 20.213	−0.350	0.727
Diastolic Pressure (mmHg)	81(75.750–91)	81.07 ± 12.472	−1.136	0.256
Smoking History	42(46.2%)	43(49.4%)	0.191	0.662
Diabetes	24(26.4%)	24(27.6%)	0.033	0.855
Fasting Blood-glucose (mmol/l)	6.03(5.085–8.235)	5.81(5.240–8.170)	−0.356	0.722
TG (mmol/l)	1.57(1.16–2.303)	1.74(1.14–2.5)	−0.856	0.392
HDL-C (mmol/l)	0.96(0.085–1.133)	0.88(0.770–1.070)	−1.901	0.057
TC (mmol/l)	4.616 ± 1.300	4.213 ± 1.048	2.233	0.027
LDL-C (mmol/l)	3.136 ± 1.047	2.770 ± 0.877	2.480	0.014
EF(%)	63(42–65)	65(63–67.25)	−3.482	0.000
Aspirin	87(95.6%)	85(97.7%)	−0.773	0.440
Clopidogrel	78(85.7%)	77(88.5%)	−0.553	0.580
Ticagrelor	1(1.1%)	2(2.3%)	−0.620	0.535
Beta blockers	55(60.4%)	45(51.7%)	−1.168	0.243
CCB	25(27.5%)	27(31.0%)	−0.521	0.602
ACE-I or ARB	47(51.6%)	30(34.5%)	−2.304	0.021
Statin	73(80.2%)	75(86.2%)	−1.064	0.287
PCI	76(83.5%)	53(60.9%)	−2.593	0.010

### Identification of real-time fluorescence quantitative PCR amplified PRMT5 gene products

The results of the real-time fluorescence quantitative PCR analysis of the RNA from the peripheral blood indicate that the amplification curve of the PRMT5 gene has an obviously smooth “S-shape”. The dissociation curve has a single dissociation peak, and the amplified product has a relatively high degree of specificity.

### Comparison of the relative expression level of the PRMT5 gene at the mRNA level between the AMI group and stable CAD group

**Fig. 1:**
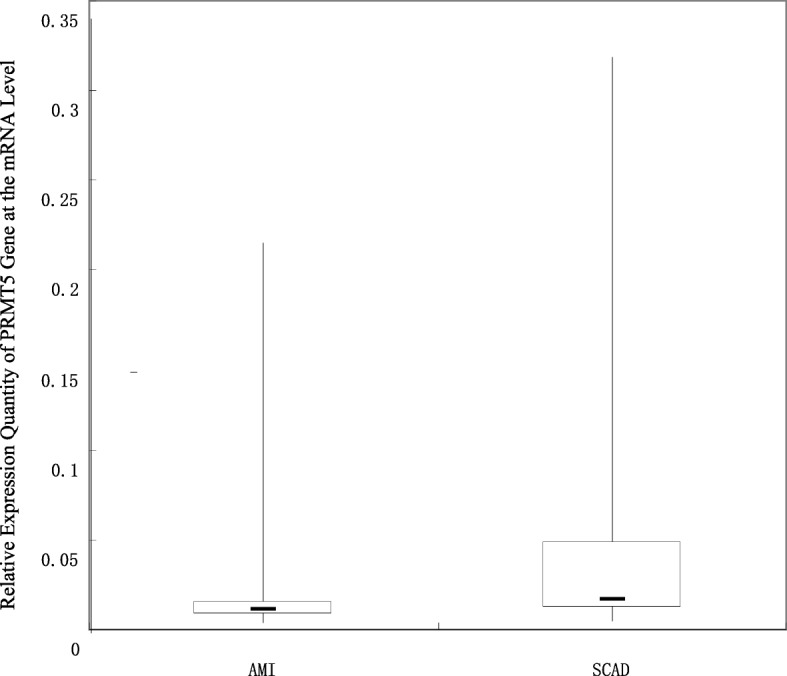
Comparison of the Relative Expression Level of the PRMT5 Gene at the mRNA Level between the AMI Group and Stable CAD Group

The ΔCt value of each sample acquired through RT-PCR represents the mean value of 3 repeated measurements of each sample. The results indicate that the 2^-△CT^ in the AMI Group is 0.013 (0.008–0.156) and that the 2^-△CT^ in the stable CAD Group is 0.017 (0.012–0.044), and this difference between the two groups is significant (Z = − 4.813, *P* = 0.000). In the peripheral blood from the AMI Group, at the mRNA level, the expression level of the PRMT5 gene is significantly lower than that in the control group, and the relative expression level of the PRMT5 gene in the AMI Group is 0.76 times that in the stable CAD Group. See Fig. 1 for details.

### Analysis of the results of the expression of the PRMT5 gene at the protein level in peripheral blood

**Fig. 2:**
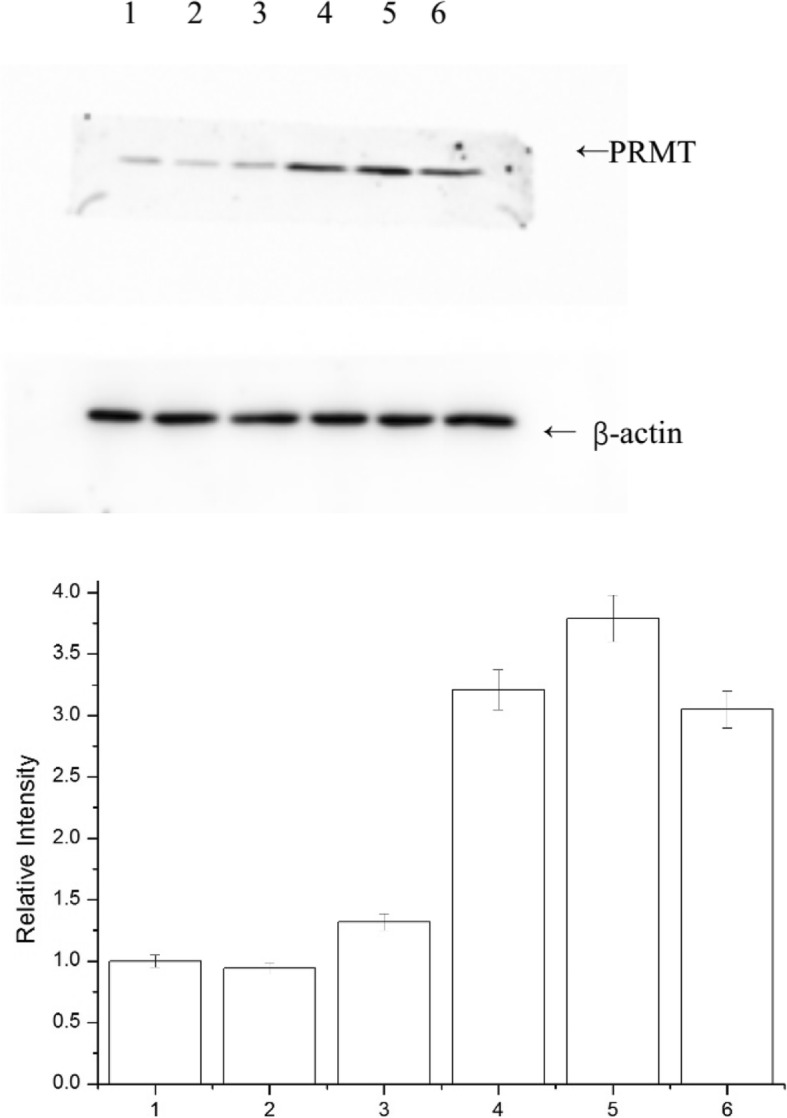
Comparison of the PRMT5 Expression Level at the Protein Level. In the AMI Group, the Sample Numbers are 1, 2 and 3, and in the Stable CAD Group, the Sample Numbers are 4, 5 and 6

In this research, β-actin served as a reference gene based on which the test of the peripheral blood from the patients at the protein level was conducted. The results of the Western blot analysis indicate that there is no obvious difference in the expression of the β-actin gene at the protein level between the AMI Group and the stable CAD Group, and there is a significant difference in the expression of the PRMT5 gene at the protein level. Compared to the stable CAD patients, the AMI patients have a low expression of PRMRT5 in their peripheral blood at the protein level, and the expression of PRMRT5 in the AMI Group at the protein level is 0.326 times that in the stable CAD Group. See Fig. 2 for details.

### Analysis of the correlation between expression levels of the PRMT5 gene and Patient characteristics

The data from these groups indicate that there was a difference in the expression level of the PRMT5 gene at the mRNA level and the levels of serum TC and LDL-C between the AMI Group and the stable CAD Group. Further analysis was conducted to analyse whether the expression level of the PRMT5 gene at the mRNA level is correlated with the serum TC and LDL-C levels. All included research subjects were divided into the following groups based on specific criteria related to the blood lipid level [23]: normal TC level group (< 5.18 mmol/L), increased TC level group (≥5.18 mmol/L), normal LDL-C level group (< 3.37 mmol/L) and increased LDL-C level group (≥3.37 mmol/L). The relative expression level of the PRMT5 gene at the mRNA level in each research subject was expressed as 2^-△Ct^, based on which the correlation between each group and the expression level of the PRMT5 gene was assessed. The results indicate that there is no difference in the expression level of the PRMT5 gene at the mRNA level between the normal TC level group and the increased TC level group (*P* = 0.363); there is no difference in the expression level of the PRMT5 gene at the mRNA level between the normal LDL-C level group and the increased LDL-C level group (*P* = 0.568); there is no difference in the expression level of the PRMT5 gene at the mRNA level between the ACEI/ARB group and the ACEI/ARB group (*P* = 0.177)The expression level of the PRMT5 gene at the mRNA level is irrelevant to the EF value of the patients (*P* = 0.993). There is no correlation among the PRMT5 gene, the EF value of the patients, ACEI/ARB, and the levels of serum TC and LDL-C. See Table 3 for details regarding the results.

**Table 3:** Analysis on correlation between expression quantity of PRMT5 gene and patients characteristics

Group	Number of members	Relative expression quantity of PRMT5	Z	*P*
The Group with Normal TC	133	0.014(0.009–0.024)	−0.910	0.363
The Group with Increased TC	38	0.016(0.008–0.032)		
The Group with Normal LDL-C	122	0.014(0.009–0.021)	−0.571	0.568
The Group with Increased LDL-C	49	0.014(0.008–0.036)		
The Group with Normal EF	109	0.014(0.010–0.031)	−0.009	0.993
The Group with low EF	23	0.014(0.007–0.049)		
The ACEI/ARB group	77	0.014(0.008–0.023)	−1.349	0.177
the non ACEI/ARB group	94	0.015(0.011–0.44)		

### Analysis of the correlation between the relative expression level of the PRMT5 gene at the mRNA level and serum TC, serum LDL-C and AMI using a logistic regression analysis

Based on the cut-off value of the relative expression level of the PRMT5 gene, all research subjects were divided into the high expression level group (2^-△Ct^ > 0.013) and the low expression level group (2^-△Ct^ ≤ 0.013). The correlations between the expression level of the PRMT5 gene at the mRNA level and serum TC, serum LDL-C and AMI were analysed by using a stepwise binary logistic regression analysis. The results indicate that a low expression level of the PRMT5 gene serves as an independent risk factor for AMI. Compared with the risk of AMI in the high PRMT5 gene expression group, the risk of AMI in the low PRMT5 gene expression group was increased by 5.472 times. However, the increase in the serum TC and LDL-C levels does not serve as an independent risk factor for myocardial infarction. See Table 4 for details.

**Table 4:** Result of logistic regression analysis on independent risk factors of AMI

	Regression Coefficient	Standard Error	Wald	P Value	OR Value	95% CI
Low Expression of PRMT5	1.700	0.340	24.964	0.000	5.472	2.809–10.657
TC Increase	−0.064	0.626	0.010	0.919	0.938	0.275–3.202
LDL-C Increase	0.785	0.574	1.870	0.171	2.193	0.712–6.758

### Analysis of the correlation between the relative expression level of the PRMT5 gene and the severity of coronary artery lesions

Based on the results of the coronary angiography, the severity of the coronary artery lesion in each research subject in the AMI Group was scored according to the Gensini scoring system [24]. In patients with ACS, the Gensini score provides more valuable prognostic information regarding the cardiovascular risk than either the Leaman or ACC/AHA score [25]. The higher the score, the more severe the coronary stenosis. The result of the Gensini scoring in the AMI Group is 42 (19.5–84.75). A double-variable correlation analysis of the relative expression level of the PRMT5 gene and the Gensini score was conducted, and the results indicate that there is a significant negative correlation between the relative expression level of the PRMT5 gene and the Gensini score (rs = − 0.205, *P* = 0.015), namely, the lower the expression level of the PRMT5 gene at the mRNA level, the higher the Gensini score, and the more severe the coronary atherosclerosis.

### Correlation between the relative expression level of the PRMT5 gene and Cardiac troponin I (TnI)

In the AMI group, the cardiac troponin test result is 0.52 (0.115–8.252) ng/ml. The concentration of serum troponin I (TnI) reflects the area of AMI. The results of the double-variable correlation analysis of serum TnI and the relative expression level of the PRMT5 gene indicate that there is no correlation between the expression level of the PRMT5 gene in peripheral blood and the serum TnI concentration (rs = − 0.125, *P* = 0.413), indicating that there is no correlation between the expression level of PRMT5 at the mRNA level and the area of AMI.

### Correlation between the relative expression level of the PRMT5 gene and Interval from time of onset of AMI to time of blood collection

The incipient long-time (≥20 min) rest pain of patients with AMI during the prior week was considered the time of onset, and the interval from the onset of AMI in the patients in the AMI Group to the time of blood collection was considered the time interval of occurrence; the result was 24 (9–54) h. The results of the analysis of the correlation between the relative expression level of the PRMT5 gene and the time interval of occurrence of AMI indicate that the expression level of the PRMT5 gene in peripheral blood is irrelevant to the time interval of occurrence of AMI (rs = − 0.146, *P* = 0.211).

## Discussion

In this research, we discovered that compared to the stable CAD patients, the AMI patients had a low expression of the PRMR5 gene in their peripheral blood at both the mRNA and protein levels. Protein arginine methyltransferases (PRMTs) can catalyse the methylation of arginine and participate in many important cellular processes that are closely correlated to the occurrence, progression and invasion of tumours, T-lymphocyte activation and hepatic gluconeogenesis [19]. PRMT5 performs many functions in the human body, can regulate the growth and transformation of cells [26] and is correlated with various types of tumours. It is possible that the expression of the PRMT5 gene in pulmonary epithelial cells represents the first step in the promotion of the occurrence of lung adenocarcinoma [27] and can promote proliferation in breast cancer cells [28]. However, no research has investigated the correlation between PRMT5 and cardiovascular diseases. Four genes in the PRMT family, including CARM1, PRMT1, PRMT5, and PRMT6, are considered related to the inflammatory response [19]. Inflammatory activity plays an important role in all phases of the progression of coronary heart disease, including thrombosis and development, and thrombosis is the cause of 80% of all sudden cardiac deaths [29]. PRMT5 may play a role in regulating the on-off action of the inflammatory response in the endothelium [19], which plays a role in regulating the inflammatory response in the endothelium by mediating the methylation of HOXA9 and regulating histone H4R3, which plays a role in gene silencing [30, 31]. PRMT5 can also participate in the immune response and plays a key role in regulating the inflammatory response by mediating NF-kB [19].

Currently, it is believed that during the progression from stable CAD to AMI, the inflammatory response plays an important role, and the PRMR5 gene serves as an important gene regulating response. Therefore, we reasonably speculate that the PRMR5 gene can promote the progression from stable CAD to AMI.

The results of this research indicate that not only was the PRMT5 gene differentially expressed but also that the levels of serum TC and LDL-C differed between the two groups based on the data. Through further analysis, we verified that the abnormal expression level of the PRMT5 gene is irrelevant to the levels of serum TC and LDL-C. Although it is generally believed that LDL can deposit at the subendothelium by integrating proteoglycan and be easily phagocytized by foam cells [32] the progression of coronary atherosclerosis is further promoted [33]. In addition, numerous studies have indicated that lipid-lowering therapy can reduce the risk of AMI among patients with coronary heart disease and increase their survival rate [34, 35]. However, some studies have indicated that compared to stable CAD patients, AMI patients have relatively lower levels of serum TC and LDL-C. The highest degree of reduction in the levels of serum TC and LDL-C occurs during the period between 4 and 12 days after the occurrence of AMI, and the levels of serum TC and LDL-C are negatively correlated with the stability of atherosclerotic plaque, but the data from these groups in this research indicate that compared to the stable CAD patients, the AMI patients have higher levels of serum TC and LDL-C, which is inconsistent with previously reported results. However, the data of these groups in this research suggest that there is no correlation between the PRMT5 gene and the levels of serum TC and LDL-C, indicating that the PRMT5 gene does not promote coronary atherosclerosis and atheromatous plaques and, thus, causes AMI by influencing the levels of serum TC and LDL-C.

In this research, the results of the binary logistic regression analysis indicate that a low expression of the PRMT5 gene serves as an independent risk factor of the progression from stable CAD to AMI in patients and causes the risk of AMI to increase by 5.472 times. The increase in the serum TC and LDL-C levels is not a risk factor for progression from stable CAD to AMI in patients. However, the mechanism of the progression from atherosclerotic heart disease to AMI is not simple lipid accumulation and is realized through the vascular inflammatory response to a larger degree [33]. Coronary heart disease is a disease caused by multiple responses, and inflammation plays an important role in the occurrence and progression of each phase and the final plaque rupture [32]. By promoting the secretion of matrix metalloproteinase by plaques, which causes the degradation of extracellular matrix protein and weakens the fibrous cap, inflammation can further result in plaque rupture and thrombosis [36] and promote the occurrence of myocardial infarction. Therefore, via its function of regulating the on-off action of inflammation, the PRMT5 gene may serve as an important susceptibility gene for the occurrence of AMI.

The results of this research also indicate that the relative expression level of the PRMT5 gene is correlated to the Gensini score, which indicates the severity of coronary artery lesions. Gensini scoring can be used to measure the severity of coronary artery lesions by adopting the method of quantitative coronary angiography in which different weights are adapted for different degrees of severity, and the result of the scoring is mainly correlated to the degree of coronary artery stenosis and scope of blood supply [24], namely, the lower the relative expression level of the PRMT5 gene, the higher the Gensini score, and the more severe the coronary artery lesion. Therefore, based on this research, we speculate that the expression level of the PRMT5 gene is correlated with the severity of coronary artery lesions.

It has been verified through the analysis of the correlation between the expression level of the PRMT5 gene and cardiac troponin I (TnI) that the expression level of the PRMT5 gene is irrelevant to the level of cardiac troponin, but the level of cardiac troponin can reflect the scope of myocardial infarction; thus, the expression level of the PRMT5 gene may be irrelevant to the scope of myocardial infarction. Meanwhile, the expression level of the PRMT5 gene is irrelevant to the time interval of the occurrence of AMI. The time interval of occurrence refer to the interval from the onset of AMI in patients to the time of blood collection. Therefore, the variation in the scope of AMI is not accompanied by a decrease in the expression level of the PRMT5 gene, and the variation in the time interval of the occurrence of AMI is not accompanied by a decrease in the expression level of the PRMT5 gene. Therefore, we speculated that if low PRMT5 expression is a consequence of AMI, it should be correlated to the concentration of cardiac troponin I (TnI) and time interval of the occurrence of AMI. If low expression of PRMT5 leads to AMI, it should also have led to a change in the trend of cardiac troponin I (TnI) and time interval of the occurrence of AMI.

Therefore, based on the results of this research, we can speculate that the low expression of the PRMT5 gene may have an influence on coronary artery lesions by regulating the inflammatory response, thus promoting the occurrence of AMI.

Certainly, to clarify whether PCI influence level of the PRMT5 gene at the mRNA level or whether the low expression of the PRMT5 gene is a cause of AMI, it is still necessary to design forward-thinking studies.

## Conclusion

Compared to stable CAD patients, AMI patients have a lower expression of the PRMT5 gene in their peripheral blood. Patients who have low PRMT5 gene expression in their peripheral blood are more likely to suffer from AMI than those with stable CAD. The low expression of the PRMT5 gene serves as an independent risk factor for the occurrence of AMI.

## Abbreviations

AMI: acute myocardial infarction
MI: myocardial infarction
PRMTs: protein arginine methyltransferases
SCAD: stable coronary artery disease
stable: CAD stable coronary artery disease
STEMI: ST-segment elevation myocardial infarction.
NSTEMI: Non-ST-segment elevation myocardial infarction
PCI: percutaneous coronary intervention
CABG: coronary artery bypass grafting
BMI: body mass index
TG: triglyceride
HDL-C: high-density lipoprotein cholesterol
TC: total cholesterol
LDL-C: low-density lipoprotein cholesterol
TnI: troponin I
ACE: angiotensin-converting enzyme
ARB: angiotensin receptor blocker
CCB: calcium channel blockers
